# The *N–*Terminal Tail of hERG Contains an Amphipathic α–Helix That Regulates Channel Deactivation

**DOI:** 10.1371/journal.pone.0016191

**Published:** 2011-01-13

**Authors:** Chai Ann Ng, Mark J. Hunter, Matthew D. Perry, Mehdi Mobli, Ying Ke, Philip W. Kuchel, Glenn F. King, Daniela Stock, Jamie I. Vandenberg

**Affiliations:** 1 Molecular Cardiology and Biophysics Division, Victor Chang Cardiac Research Institute, Darlinghurst, New South Wales, Australia; 2 School of Molecular Biosciences, University of Sydney, Sydney, New South Wales, Australia; 3 Institute for Molecular Bioscience, The University of Queensland, St Lucia, Queensland, Australia; 4 Structural and Computational Biology Division, Victor Chang Cardiac Research Institute, Darlinghurst, New South Wales, Australia; 5 St Vincent's Clinical School, University of New South Wales, Sydney, New South Wales, Australia; University of South Florida College of Medicine, United States of America

## Abstract

The cytoplasmic *N–*terminal domain of the human ether–a–go–go related gene (hERG) K^+^ channel is critical for the slow deactivation kinetics of the channel. However, the mechanism(s) by which the *N–*terminal domain regulates deactivation remains to be determined. Here we show that the solution NMR structure of the *N–*terminal 135 residues of hERG contains a previously described Per–Arnt–Sim (PAS) domain (residues 26–135) as well as an amphipathic α–helix (residues 13–23) and an initial unstructured segment (residues 2–9). Deletion of residues 2–25, only the unstructured segment (residues 2–9) or replacement of the α–helix with a flexible linker all result in enhanced rates of deactivation. Thus, both the initial flexible segment and the α–helix are required but neither is sufficient to confer slow deactivation kinetics. Alanine scanning mutagenesis identified R5 and G6 in the initial flexible segment as critical for slow deactivation. Alanine mutants in the helical region had less dramatic phenotypes. We propose that the PAS domain is bound close to the central core of the channel and that the *N–*terminal α–helix ensures that the flexible tail is correctly orientated for interaction with the activation gating machinery to stabilize the open state of the channel.

## Introduction

The human ether–a–go–go related gene (*hERG*) encodes K_v_11.1, the pore forming subunit of the rapidly activating delayed rectifier K^+^ channel (I_Kr_) [1]. Reduction of hERG channel activity either by drugs [2] or genetically inherited mutations [3] results in prolongation of the QT interval on the surface electrocardiogram and a markedly increased risk of arrhythmias and sudden cardiac death [4]. hERG channels are tetrameric with each subunit containing cytoplasmic *N–* and *C*–terminal domains and six transmembrane domains. The fifth and sixth transmembrane domains along with an intervening pore helix from each of the four subunits surrounds the ion conducting pore [5]. In addition, a cyclic nucleotide binding domain (cNBD) immediately *C*–terminal to the pore domain is thought to contribute to the stabilization of the tetrameric structure [6]. Conversely, the cytoplasmic *N–*terminus of each subunit contains a Per–Arnt–Sim (PAS) domain (residues 26–135) [7], [8] that is stable as a monomer and interacts with the remainder of the channel thereby regulating the kinetics of channel opening and closing [7], [9], [10].

In hERG, *N–*terminal deletions that remove the PAS domain (Δ2–373 [11], Δ2–354 [12], [13], and Δ2–138 [7]) significantly enhance the rate of deactivation of the channel. Further, the *N–*terminal domain (residues 1–136) is able to restore deactivation gating in *N–*terminally truncated hERG [7],[9]. However, deletions within the short *N–*terminal tail that precedes the PAS domain (residues 1–25) also result in significantly faster rates of deactivation [7], [12], [13]. Moreover, application of a peptide corresponding to the *N–*terminal 16 residues can slow the deactivation kinetics of channels with most of the *N–*terminus deleted (Δ2–354, [12]).

To clarify the role of the *N–*terminal tail domain in hERG K^+^ channel deactivation, we determined the solution state structure of a construct encompassing both the PAS domain and the *N–*terminal tail. We show that the tail contains an amphipathic α–helical region from T13 to E23. Deletion of either the initial unstructured segment (Δ2–9) or replacement of the amphipathic α–helical region with a flexible linker resulted in faster deactivation, suggesting that both regions are necessary but neither is sufficient to permit normal deactivation. Alanine scanning of the *N–*terminal tail indicated that residues R5 and G6 are involved in critical interactions that stabilize the open state of the channel. Although the majority of alanine mutations in the amphipathic α–helical region did not have a significant effect on the rate of deactivation, both I19A and R20A showed an enhanced rate of deactivation. This suggests that the α–helix may act as a spacer, rather than being involved in critical specific interactions with other domains of the channel.

## Materials and Methods

### Protein expression

The PAS domain (residues 1 to 135) was expressed as an *N–*terminal cleavable glutathione–S–transferase (GST) fusion in *E. coli* C41 strain after overnight induction at 20°C. A freeze/thaw method was used to lyse the cells with 20 mM Tris buffer containing 5 mM 2–mercaptoethanol, 0.1% v/v Tween 20, and 150 mM NaCl. The lysate was incubated with glutathione beads (GE Healthcare, Amersham, UK) for 3 h and the protein eluted by TEV protease digestion overnight at 4°C. The protein was concentrated and passed through a Superdex 75 column (GE Healthcare), equilibrated in 10 mM HEPES pH 6.9, 150 mM NaCl, 5 mM *N–*octyl–D–glucoside (OG) (Anatrace Inc., Maumee, OH, USA) and 3 mM *tris*(2–carboxyethyl)phosphine (TCEP). The purified PAS domain eluted as a single peak at the expected molecular size for a monomer (as previously described [7]). The ^13^C/^15^N double–labelled PAS domain was produced by substituting the nitrogen and carbon sources in bacterial growth medium with ^15^N*–*enriched NH_4_Cl and ^13^C–enriched glucose, respectively.

### Sample preparation and NMR spectroscopy

The NMR sample consisted of 0.21 mM ^13^C/^15^N PAS domain protein in solution containing 10 mM HEPES, 3 mM TCEP, 5 mM OG, and 7% D_2_O, at pH 6.9. All NMR experiments were performed on a Bruker Avance II 900 MHz NMR spectrometer at 298 K. 2D ^1^H–^15^N HSQC, 3D ^1^H–^15^N NOESY and 3D ^1^H–^13^C NOESY data were acquired using traditional methods while 3D HNCO, HNCA, HN(CO)CA, HNCACB, CBCA(CO)NH, C(CO)NH, H(CCO)NH and HCCH–TOCSY data were acquired using a non*–*uniform sampling method and maximum entropy reconstruction [14]. The sample was buffer–exchanged in D_2_O before acquiring 3D HCCH–TOCSY and ^1^H–^13^C NOESY NMR spectra.

### NMR chemical shift assignment and structure calculations

All NMR spectra were analysed using XEASY3 [15]. Sequence–specific backbone assignments were made using 3D HNCO, HNCA, HN(CO)CA, HNCACB and CBCA(CO)NH data. Side–chain chemical shift assignments were made using 3D (H)CC(CO)NH–TOCSY, H(CC)(CO)NH–TOCSY and HCCH–TOCSY data. A total of 2634 distance constraints were derived from 3D ^1^H–^15^N and ^1^H–^13^C NOESY data, 24 hydrogen bond constraints were derived from the ^1^H–^13^C NOESY data (based on amide protons that were still observable afte exchange of the sample into D_2_O buffer), and 178 dihedral angle constraints (φ,ψ) were derived from TALOS [16]. The error range used in the structure calculations were set to twice the standard deviation estimated by the program. Automated NOE assignment and structure calculations were performed using the program CYANA v2.1 [17]. An ensemble of the 20 structures with the lowest target function values was chosen to represent the solution structure of the protein. Energy minimization of these structures was performed using the program AMBER 10 [18]. The generalized Born (GB) solvent model was used for the final energy minimization using the distance constraints from the CYANA calculation. The energy–minimized structures were validated using the PSVS server [19] and deposited in the PDB [20] under the accession code 2L0W. Chemical shift assignments were also deposited in the BioMagResBank under accession code 17066. Secondary structure elements were predicted using Talos+ [21].

### Electrophysiology

HERG cDNA (a gift from Dr Gail Robertson, University of Wisconsin) was subcloned into a pBluescript vector containing the 5′ untranslated region (UTR) and 3′ UTR of the *Xenopus laevis* β–globin gene (a gift from Dr Robert Vandenberg, University of Sydney). Mutagenesis was carried out using the Quickchange mutagenesis method (Agilent Technologies, CA, USA) and confirmed by DNA sequencing. Wild–type (WT) and mutant channel cDNAs were linearized with BamHI and cRNA transcribed with T7 RNA polymerase using the mMessage mMachine kit (Ambion, city, TX, USA).

*Xenopus laevis* oocytes were prepared as previously described [22]. Stage V and VI oocytes were isolated, stored in tissue culture dishes containing ND96 (in mM: KCl 2.0, NaCl 96.0, CaCl_2_ 1.8, MgCl_2_ 1.0 and HEPES 5.0) supplemented with 2.5 mM sodium pyruvate, 0.5 mM theophylline and 10 µg mL^−1^ gentamicin, adjusted to pH 7.5 with NaOH and incubated at 18°C. All experiments were approved by the Garvan/St Vincent's Animal Ethics Committee (Approval ID 08/34).

*Xenopus laevis* oocytes were injected with 5–10 ng cRNA and incubated at 18°C for 24–48 h prior to electrophysiological recordings. All experiments were undertaken at room temperature (21–22°C). Two–electrode, voltage–clamp experiments were performed using a Geneclamp 500B amplifier (Molecular Devices Corp, Sunnyvale, CA, USA). Glass microelectrodes had tip resistances of 0.3–1.0 MΩ when filled with 3 M KCl. Oocytes were perfused with ND96 solution (see above). In all protocols a step depolarization of +20 mV from the holding potential of −90 mV was applied at the start of each sweep to enable off–line leak–current subtraction. We assumed that the current leakage was linear in the voltage range −160 to +40 mV. Data acquisition and analysis were performed using pCLAMP software (Version 9.2, Molecular Devices Corp, Sunnyvale, CA, USA) and Excel software (Microsoft, Seattle, WA, USA). All parameter values were estimated as mean ± standard error of the mean (SEM) for *n* experiments, where *n* denotes the number of different oocytes studied for each construct.

Isochronal activation curves were measured using standard tail current analysis [1]. Cells at a holding potential of −90 mV were subjected to 4–s depolarizing steps to voltages in the range −70 to +50 mV before stepping the voltage to −70 mV where tail currents were recorded. Tail current data were normalized to the maximum current value (*I*_max_) and fitted with a Boltzmann expression:
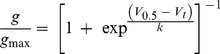
(1)where *g/g_max_* is the relative conductance, *V*_0.5_ is the half–activation voltage, *V*_t_ is the test potential and *k* is the ‘slope factor’. Alternatively, the data were fitted with the thermodynamic form of the Boltzmann expression:
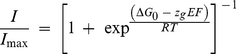
(2)where Δ*G*_0_ is the work done at 0 mV, *z*_g_ is the effective number of gating charges moving across the membrane electric field, *E*. F, is Faraday's constant, R is the universal gas constant and T is the absolute temperature. Equations (1) and (2) are equivalent, however from Equation (2) we can calculate the effect of mutations on changes in the chemical potential (Δ*G*_0_) and electrostatic potential (–*z*_g_ *E*F) that drives activation and deactivation of the channel.

To measure rates of deactivation, oocytes were depolarized from a holding potential of −90 mV to +40 mV for 1 s to fully activate the channels; they were then repolarized to potentials in the range −50 to −160 mV. A double exponential function was fitted to the decaying portion of tail currents. In order to compare rates of deactivation for different mutants at comparable driving forces, the voltages in the range −60 to −160 mV were converted to electrochemical driving forces (–*z*_g_ *E*F) as defined in Equation (2).

## Results

**Figure 1:**
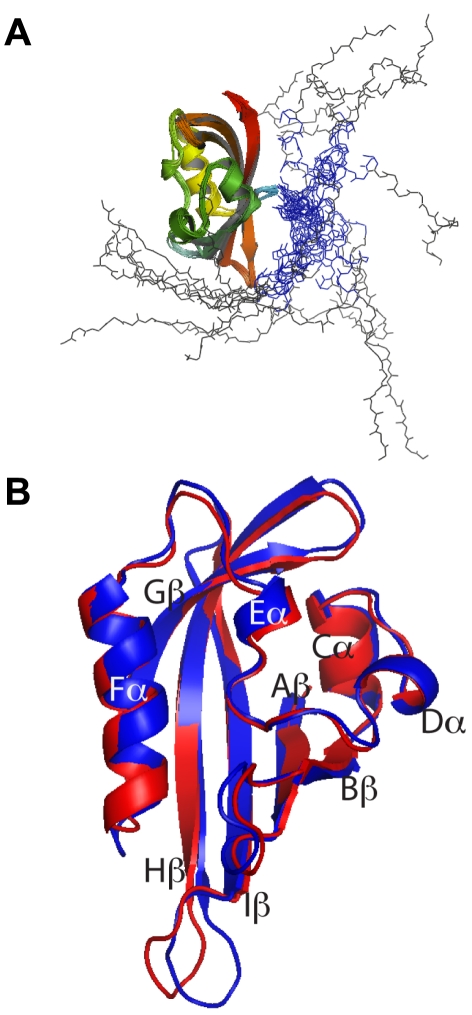
NMR structure of hERG *N*–terminal domain. Ensemble of 20 structures of the hERG *N*–terminal domain (residues 1–135) shown in panel A. The PAS domain is shown as a ribbon representation with a color ramp from green at the *N*–terminus to red at the *C*–terminus. The *N*–terminal α–helix (T13–E23) is shown in blue and the remaining residues (M1–N12) are shown in black. B. Superposition of the lowest–energy NMR structure (blue) on the previously determined crystal structure (1BYW, red [7]) yields a backbone RMSD of 0.78 Å. The *N*–terminal tail (M1–Q25) was excluded in this superposition. Helices and sheets are labelled using the schema devised by Moglich *et al*. [30].

The solution structure of hERG 1-135 domain was well folded with dispersed peaks in the ^1^H-^15^N-HSQC (Fig. S1). More than 97% of the backbone amides were assigned, the exceptions being A40 and R77. 75% of the sidechain N–H from arginine, asparagine and glutamine were assigned; the exceptions were R4, R5, R73, R76, R77 and Q84. All resonances of Hα and Hβ atoms were assigned except for Hβ of R4 and R5. All Cα, Cβ and C were assigned except for the carbonyls of R76 and V113. In addition, more than 90% of the remaining sidechain proton and carbon resonances were assigned. These assignments allowed a total of 2634 distance constraints (Table 1) to be unambiguously derived and these were used in combination with the dihedral angle and hydrogen–bond constraints to calculate the solution structure of the *N–*terminal 135 residues of the hERG K^+^ channel (Fig. 1). The RMSDs of all backbone and heavy atoms excluding the *N–*terminal tail (S26 to K135) were 0.40 and 1.10 Å, respectively.

**Table 1:** Structural statistics of the final 20 ensemble of PAS domain in solution.

Parameter		Ensemble
Total number of NOE constraints used:		2634
Short range	| i – j | < = 1	1259
Medium range	1< | i – j | < 5	498
Long range	| i – j | > = 5	877
NOE constraints per residue (135 residues)		19
Distance constraints for hydrogen-bonds:		24 (48 including upper and lower constraints)
TALOS backbone dihedral angle constraints		89 (φ), 89 (ψ)
Coordinate r.m.s.d. (Å) for all residues(S26-K135) exluding N-terminal tail		
Average backbone r.m.s.d. to mean		0.40
Average heavy atom r.m.s.d. to mean		1.10
Ramachandran assessments *		
MolProbity (%)		
Favored regions		96.9 (95.8)
Allowed regions		2.9 (3.8)
Disallowed regions		0.2 (0.4)

*ordered residues (F14-F22;R27-R62;T65-E134) and all residues (M1-K135), respectively.

Statistics highlighting the extremely high precision and stereochemical quality of the ensemble of hERG PAS domain and *N*–terminal tail structures are shown in Table 1. The average MolProbity score of 1.55 places the ensemble in the 94th percentile relative to all other structures ranked by MolProbity [23]. The high stereochemical quality of the ensemble stems from a complete absence of bad close contacts, high Ramachandran plot quality (95% of residues in the most favored region), and a very low percentage of unfavorable sidechain rotamers. During the automated NOESY assignment/structure calculation process the CANDID module of CYANA assigned 86% of all NOESY crosspeaks to give an average of 19 NOE constraints per residue.

The NMR solution structure for the segment from residues S26 to K135 was almost identical to the crystal structure of the same region (Fig. 1B). The average RMSD of this NMR ensemble relative to the crystal structure (1BYW) was less than 1 Å. The only significant discrepancy was observed for the loop between Hβ and Iβ, a region previously shown to be highly dynamic in a molecular dynamics study [24].

**Figure 2:**
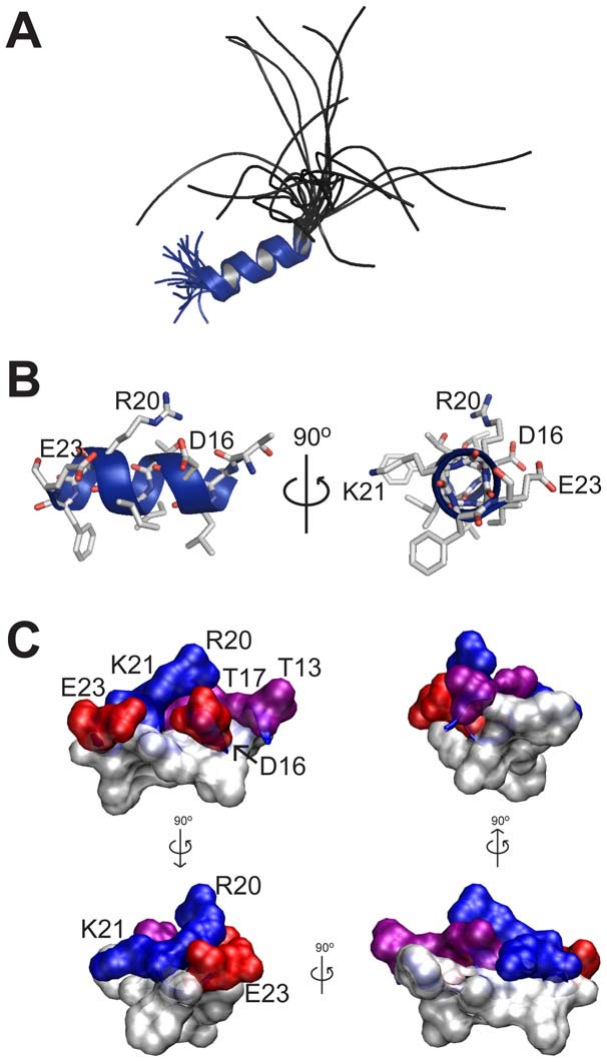
Structure of *N*–terminal tail domain. hERG residues 13–23 form an amphipathic α–helix. (A) Ensemble of the 20 lowest energy NMR structures superimposed over the backbone of residues T13 to E23 (blue). Only residues 1–25 are shown. The first 12 residues (M1 to N12, black) are disordered in solution. (B) Distribution of charged residues in the *N–*terminal helical segment. (C) Surface representations of the *N*–terminal helix. Each view was rotated 90° anti–clockwise on the y–axis. Positively charged residues (R20 and K21; blue), negatively charged residues (D16 and E23; red) and neutral polar residues (T13 and T17; purple) are located on the same side of the helix while non–polar residues (white), with the exception of I19, are located on the opposite face.

In addition to the structure of the PAS domain (residues S26 to K135), the NMR solution structure revealed a randomly distributed α–helical tail (blue lines in Fig. 1A), in particular residues T13 to E23 (Fig. 2A) with a backbone RMSD of 0.31 Å. Despite not being able to determine a fixed conformation of the *N–*terminal tail, it was consistent with the secondary structure prediction using the NMR chemical shifts and inter–residue NOE constraints (Fig. S2).

Closer examination of the *N–*terminal helix (T13 to E23) revealed that it was amphipathic with positively charged residues (R20 and K21), negatively charged residues (D16 and E23), and polar residues (T13 and T17), located on the same side of the helix (Fig. 2B), while non*–*polar residues (F14, L15, I18, I19 and F22 but not I19) were located on the opposite face of the helix. Residues 1–9 are disordered with no clearly defined structure, while residues 10–12 adopted a turn conformation.

**Figure 3:**
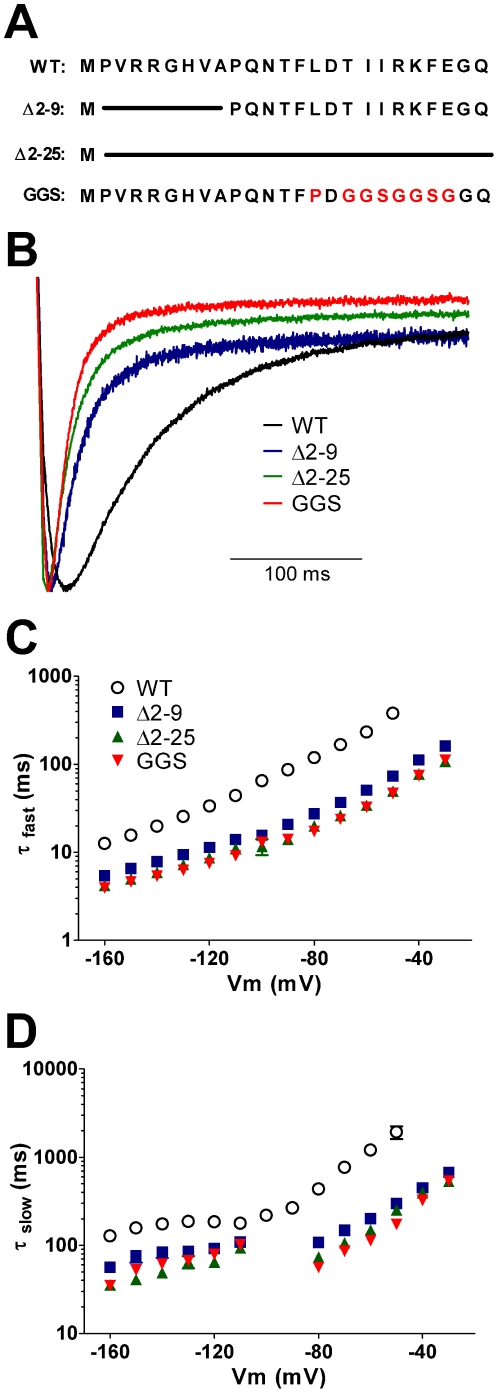
Both the unstructured tail and the α–helix of the *N*–terminal domain are required to slow deactivation kinetics of WT hERG channels. Sequence of the *N*–terminal domain in WT hERG1 channels is shown in panel A. Mutant constructs were designed to examine the effect of removing the unstructured tail (Δ2–9) or the entire *N*–terminal domain (Δ2–25), or the effect of disrupting the *N*–terminal α–helix (GGS) by replacing residues L15 and T17–E23 with P_GGSGGSG as shown in red. (B) Typical rates of deactivation observed in tail currents recorded at −120 mV following a step to +40 mV. To aid comparison current traces were normalized to peak tail current. Constructs Δ2–9 (blue), Δ2–25 (green) and GGS (red) all produced channels with faster deactivation rates than WT hERG (black). (C and D) Mean ± SEM for deactivation rates recorded over a range of voltages from −50 mV to −160 mV. Current decay associated with channel deactivation was best fitted to two exponentials, generating *τ*_fast_ (C) and *τ*_slow_ (D) for WT (open circles, *n = 11*), Δ2–9 (blue squares, *n = 14*), Δ2–25 (green triangles, *n = 5*) and GGS (red inverted triangles, *n = 10*) channels. All mutant channels enhanced the rates of both the fast and slow components of deactivation over the entire voltage range.

To study the functional significance of the α–helix in the *N–*terminal tail, we compared the effects of deleting only the initial portion of the *N–*terminus (Δ2–9), with deletion of the entire *N–*terminal tail (Δ2–25), or replacement of the α–helix with a flexible linker (denoted GGS mutant, see Fig. 3A). Typical examples of tail currents for Δ2–9, Δ2–25 and GGSmut channels (recorded at –120 mV and normalized to the peak inward current amplitude) are shown in Fig. 3B. All mutant channels showed significant enhancement of both the fast (Fig. 3C) and slow (Fig. 3D) components of deactivation over the entire voltage range studied. None of the mutants affected the relative amplitudes of the fast and slow components of deactivation at the most negative potentials. However, at less negative potentials where the fast component became less dominant, the amplitude of the fast component relative to the slow component was greater in all three mutant channels compared to WT hERG (Fig. S3).

There were small, but statistically significant, differences in the rates of deactivation between Δ2–25, Δ2–9 and GGSmut channels; e.g., at −120 mV the time constant for the fast component of deactivation was 11.4±0.3 ms (n = 14, for Δ2–9), 8.7±0.3 ms (n = 5, for Δ2–25) and 7.5±0.2 ms (n = 10, for GGS mut) compared to 33.7±1.0 ms (n = 11, for WT hERG channels).

**Figure 4:**
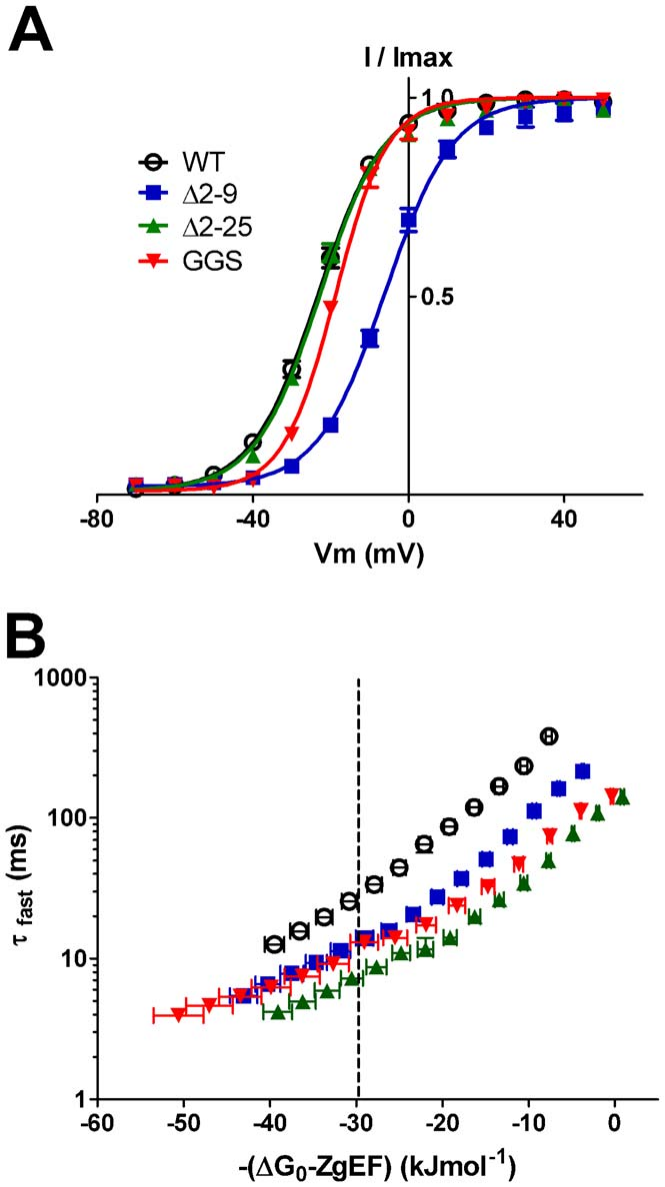
Rates of deactivation after correction for changes in activation properties. Steady–state activation curves for WT (open circles, *n = 11*), Δ2–9 (blue squares, *n = 14*), Δ2–25 (green triangles, *n = 5*) and GGS (red inverted triangles, *n = 10*) channels are shown in panel A. Peak tail currents recorded at −70 mV, following steps to a range of potentials between −70 mV to +50 mV, were normalized to *I_max_* and plotted against the preceding voltage. Data represented as mean ± SEM were fitted with Boltzmann expression (solid lines, *see Methods*). Construct Δ2–9 gave channels exhibiting a shift in the half maximal voltage for activation (*V*_0.5_) in the depolarizing direction compared to WT hERG. B. Rates of deactivation (*τ*_fast_ from Fig. 3C) are plotted against the total electrochemical driving force –(ΔG_0_–*z*_g_ *E*F) for channel deactivation. Deactivation rates taken at an equivalent driving force of −30 kJmol^–1^ (dotted line) indicate that all mutant channels deactivate faster than WT hERG (symbols as in A).

When comparing the rates of deactivation for WT and mutant channels at a single voltage, it is important to consider that changes to steady–state activation can affect the electrochemical potential for deactivation. Steady–state activation properties were determined by fitting, with a single Boltzmann expression (Equation 1), the I–V relationship of peak tail currents at −70 mV plotted against the preceding voltage step (Fig. 4). The resulting half–maximal voltage for activation (V_0.5_) of Δ2–9 channels (−5.6±0.9 mV, n = 14) was shifted in the depolarizing direction compared to WT hERG (−23.2±0.8 mV, n = 11, ANOVA p<0.05), without any change in slope. A small but statistically significant shift in activation V_0.5_ was also observed for GGSmut (−18.5±0.6 mV, n = 10) channels, while Δ2–25 channels were similar to WT hERG (Fig. 4A). The chemical (ΔG_0_) and electrostatic (–*z*_g_ *E*F) potential that drives activation was calculated by fitting the activation data with a Boltzmann function in the form of Equation 2 (data summarized in Table S1). Since changes in ΔG_0_ parallel changes in activation V_0.5_, Δ2–9 channels had a significantly smaller chemical potential for activation (–1.9±0.3 kJ mol^–1^) than WT hERG (−6.8±0.2 kJ mol^−1^, ANOVA p<0.05). To compensate for changes in electrochemical driving force, the rates of deactivation calculated at voltages between −50 mV to −160 mV were plotted against the electrochemical potential for deactivation (Fig. 4B). After correction, Δ2–9, Δ2–25 and GGSmut channels all had enhanced rates of deactivation compared with WT hERG (ANOVA p<0.05). Thus, alterations to the electrochemical driving force for deactivation could not explain the enhanced deactivation rates seen with these mutant channels.

**Figure 5:**
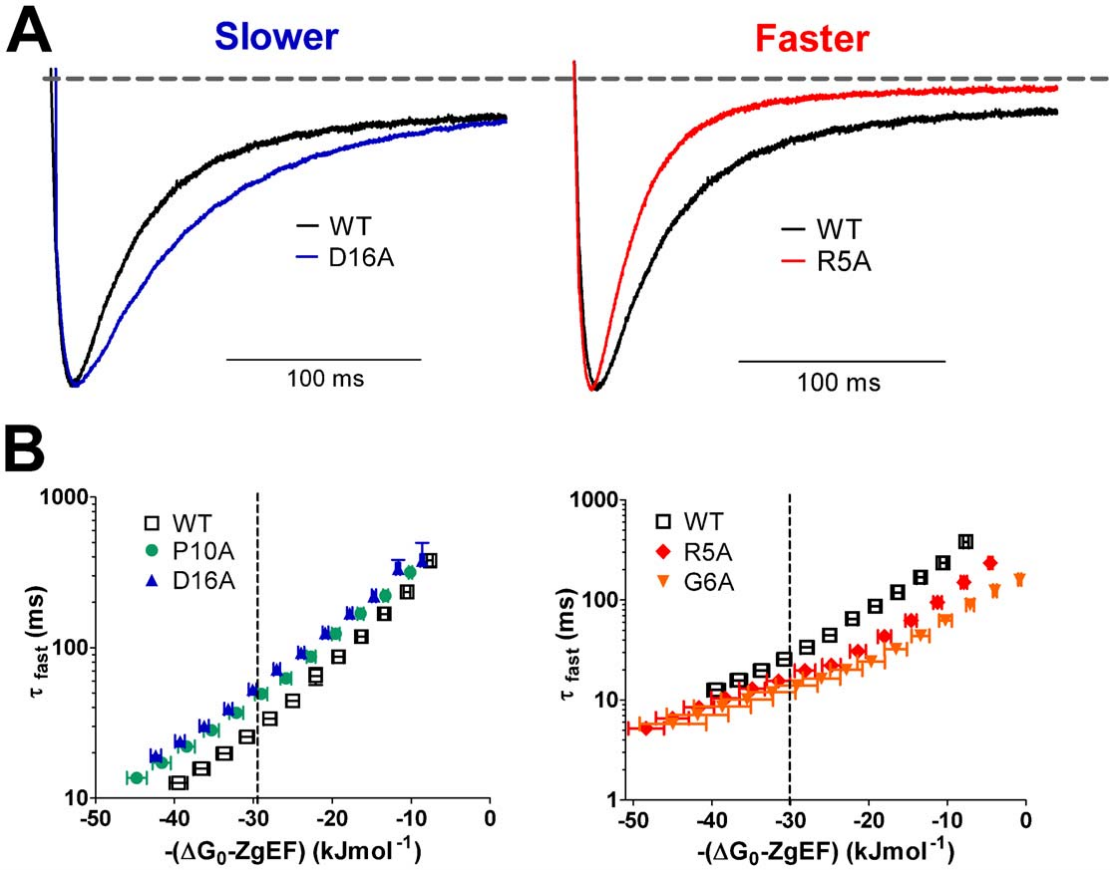
Point mutations within the *N*–terminal tail alter deactivation rates of hERG channels. Examples of point mutations that either slow (D16A, blue trace in left panel) or enhance (R5A, red trace in right panel) rates of deactivation compared to WT hERG (black traces) are shown in panel A. Rates of deactivation are represented by the decay in tail currents recorded at −120 mV following a step to +40 mV. Current traces were normalized to peak tail current to aid comparison. B. Mean ± SEM rates of deactivation (*τ*_fast_) plotted against the total electrochemical driving force –(ΔG_0_–*z*_g_ *E*F) for channel deactivation. When compared at an equivalent driving force of –30 kJmol^–1^ (dotted line) four mutant channels exhibited altered deactivation rates compared with WT hERG (open squares, *n = 11*). Two mutations, P10A (green circles, *n = 14*) and D16A (blue triangles, *n = 10*), produced channels that were slower than WT (left panel), while R5A (red diamonds, *n = 8*) and G6A mutant channels (orange inverted triangles, *n = 7*) deactivated faster than WT hERG (right panel).

**Figure 6:**
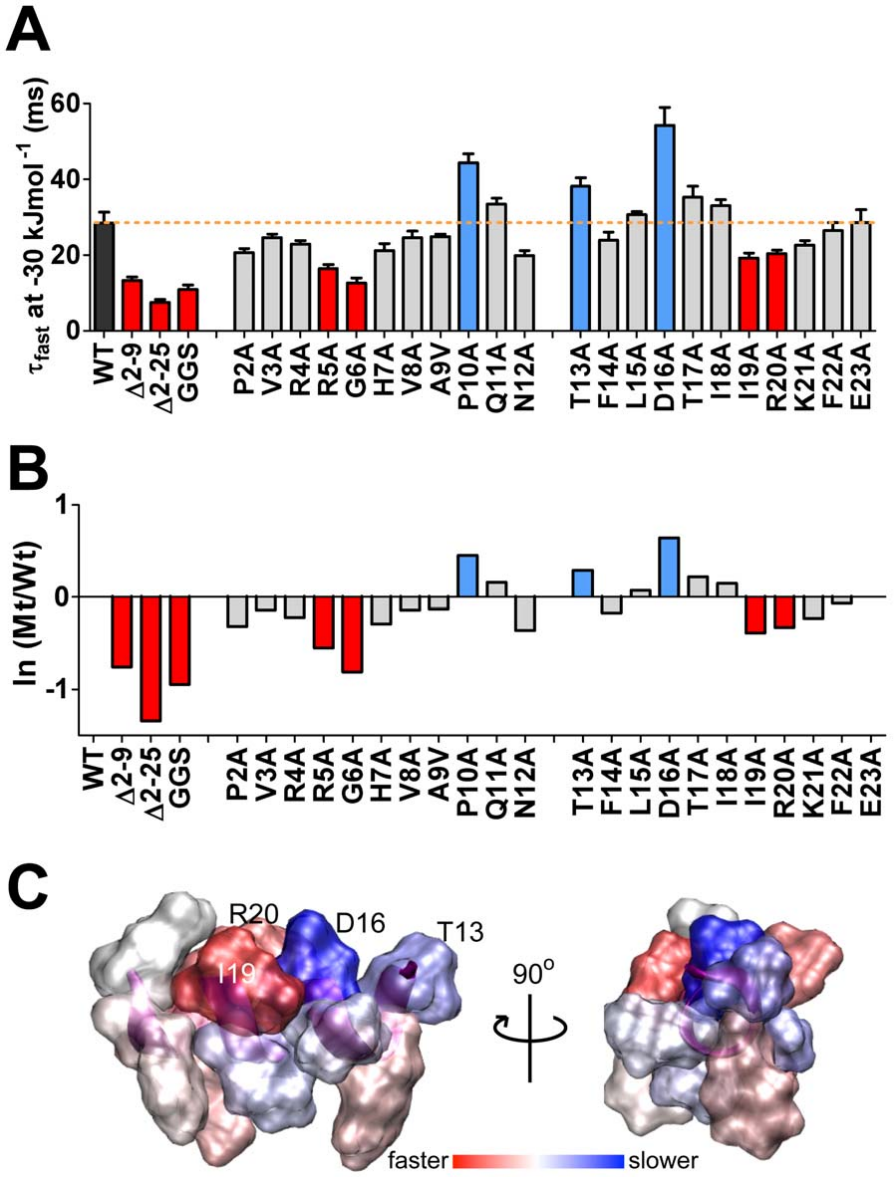
Summary comparing rates of channel deactivation for point mutations within the *N*–terminal tail. Deactivation rates (*τ*_fast_) compared at an equivalent electrochemical driving force -(ΔG_0_–z_g_ *E*F) of −30 kJmol^–1^. Data represented as mean ± SEM (panel A) or as ratio with WT hERG (panel B). Mutant constructs (Δ2–9, Δ2–25 and GGS) and point mutations (R5A, G6A, I19A, and R20A) that significantly enhanced rates of deactivation compared to WT (black bar) are shown in dark red, while point mutations (P10A, T13A and D16A) that slow deactivation are shown in dark blue (ANOVA p<0.05). C. Representation of the *N*–terminal α–helical region with residues colored depending on the severity of deactivation phenotype after mutation to alanine. Residues with the most severe alterations to deactivation rates (T13, D16, I19 and R20) clustered on the same face on the α–helix.

To probe the role of individual residues within the *N–*terminal tail, native residues from P2–E23 were individually replaced with alanine, or with valine in the case of A9. Measured rates for fast (τ_fast_) and slow (τ_slow_) components of deactivation, in addition to the relative contributions of these components are given in Table S2. At negative potentials, the fast component accounted for the majority of deactivation (>80%). This parameter was therefore used to compare WT and mutant channels. Several of the mutations introduced small (less than ±10 mV), and statistically significant shifts in the voltage dependence of channel activation when compared to WT hERG (Table S1). Accordingly, the effects of each mutation on deactivation rate were compared at an equivalent driving force of −30 kJmol^–1^ (as indicated in Fig. 5). In Fig. 6, the effect of alanine mutants on deactivation rates are classified into those that were unchanged (grey bars), faster (red bars) and slower (blue bars) compared to WT.

Typical tail currents recorded at −120 mV, as well as mean *τ*_fast_ values plotted against electrochemical driving force, are shown in Fig. 5A for two mutations (R5A and G6A) located in the unstructured *N*–terminal tail. Both the R5A and G6A mutants had significantly faster deactivation rates than the WT channel; the *τ*_fast_ at −30 kJmol^–1^ was 16.5±1.0 ms (n = 8) for R5A and 12.7±1.3 ms (n = 7) for G6A compared with 28.5±2.8 ms (n = 11) for WT (ANOVA p<0.05, Fig. 6). These data suggest that either, or both, of these residues could interact with other parts of the channel protein to slow deactivation rates in WT hERG.

Within the amphipathic *N–*terminal α–helix (residues T13 to E23), alanine mutations I19A and R20A gave channels with faster rates of deactivation when compared with WT hERG; τ_fast_ at −30 kJmol^–1^ was 19.2±1.3 ms (n = 13) for I19A and 20.4±1.0 ms (n = 20) for R20A (*vs* WT of 28.5±2.8 ms, *ANOVA,* p<0.05). Somewhat surprisingly, two mutations in the α –helical region, i.e., T13A (τ_fast_, 38.2±2.2 ms, n = 15) and D16A (τ_fast_, 54.2±4.7 ms, n = 10) exhibited slower deactivation rates than WT (ANOVA, p<0.05). Interestingly, the four residues with altered deactivation rates (T13, D16, I19, R20) lay on one face of the amphipathic helix (Fig. 6C). In addition, mutation of one residue (P10A) that lies between the amphipathic α–helix and the Δ2–9 region, also significantly slowed deactivation (44.4±2.4 ms, n = 14) compared with WT hERG (28.5±2.8 ms, ANOVA p<0.05).

## Discussion

The solution structure of hERG PAS domain determined in this study was found to be very similar to the crystal structure determined previously by Morais Cabral et al. [7] and the solution structure recently reported by Li and colleagues [25]. Our solution structure of the PAS domain (residues 26–135) superimposed very well with the crystal structure apart from the loop between Hβ and Iβ that was previously shown to be highly dynamic [24]. The major difference between the X–ray and NMR structures is that in the latter the *N–*terminal tail contained an amphipathic α–helix from residues T13 to E23. The first nine residues in the NMR structures were unstructured as were residues 24–26 that link the α–helix to the PAS domain. The remaining tail–domain residues (P10, Q11, N12) appeared to adopt a turn conformation but there were insufficient NOE constraints to be able to define it as an extension of the α–helix.

Our functional, electrophysiological data indicated that removal of either the initial unstructured segment (Δ2–9) or replacement of the α–helix with a flexible linker (GGS mutant) both produced similar phenotypes, i.e. markedly faster rates of deactivation. This is essentially the same phenotype produced by deletion of the entire *N–*terminal tail (Δ2–25, Fig. 3A, or Δ2–23 [7] or Δ2–26 [7]). These data indicate that the initial unstructured *N–*terminal segment and the α–helical region (residues 13–23) were both required, but neither alone was sufficient, for the normal slow deactivation kinetics of WT hERG channels.

Alanine scanning mutagenesis of the initial unstructured *N–*terminal segment (P2 – N12) identified R5 and G6 as the most critical residues for deactivation. Both R5A and G6A mutants had enhanced rates of deactivation that could easily explain the faster deactivation observed with deletion of residues 2–9. Within the α–helical segment two alanine mutants resulted in enhanced rates of deactivation (I19A and R20A). These mutants resulted in a smaller perturbation to deactivation than R5A and G6A, nevertheless the combined effect of all four residues may explain why deletion of residues 2–25 caused a greater enhancement of the rate of deactivation than did deletion of residues 2–9.

Whilst the deletion mutants and the GGS mutant all had faster deactivation phenotypes, three alanine mutants resulted in slowed deactivation, *i.e.,* T13A and D16A in the α–helix and P10A in the linker between the α–helix and the initial unstructured segment. It is possible that these three residues are important for ensuring that the tail does not bind too tightly to the open state of the channel. It is noted that T13 and D16 lie on the same side of the α–helix as I19 and R20, which are the only other residues in the α–helix that significantly perturbed deactivation. This suggests that this surface of the α–helix is involved in protein–protein interactions that affect the rate of deactivation. However, when we mapped the functional effect of alanine mutants onto the structure of the *N*–terminal α–helix, the residues with perturbed function did not lie along the entire length of the α–helix (Fig. 6C). We therefore suggest that in addition to providing some specific interactions the α–helix may also serve as a spacer to ensure that the flexible tail is held a predetermined distance from the PAS domain itself. Given that alanine mutants tend to stabilize α–helices it is also possible that the P10A mutant may have stabilized a longer helical domain that results in slower deactivation.

It is important to recall that the structure we have solved is an isolated domain. It is possible that the *N–*terminal tail structure reported here is more flexible than it would be in the whole, intact channel protein. Conversely, it is clear that the *N*–terminal tail interacts with another part of the channel protein to regulate deactivation and we suggest that the flexibility of the distal *N–*tail (residues 1–9) is important for its function and/or regulation.

### Model for the structural basis of deactivation gating

Deletion of the *N–*terminal tail (Δ2–25, Fig. 3A), the entire PAS domain (Δ2–138, [7]) or majority of the *N–*terminus (Δ2–354 [13], Δ2–373 [11]) all result in a very similar phenotype, i.e. approximately 5–fold faster rate of deactivation. A plausible hypothesis that explains these observations is that the PAS domain binds (with relatively high affinity) to another domain on the hERG channel and it positions the flexible *N–*terminal tail region close to the central core of the hERG channel where it binds and unbinds sufficiently rapidly to modulate the rate of deactivation. The region(s) of the channel where the PAS/*N–*terminal α–helix and the flexible *N–*terminal domains bind remain to be determined. Two obvious candidates are the S4–S5 linker [25], a part of the channel known to be critical for regulation of deactivation gating [12], [13], [26] and the C–linker + cyclic–nucleotide binding domain, as mutations in this domain modulate the kinetics of deactivation [27], [28], [29]. Li and colleagues showed that the PAS domain can bind to the S4–S5 linker, however these studies were perfomed with an isolated S4–S5 peptide fragment and need to be confirmed in studies involving either the entire channel protein or at least larger domains. Similarly, testing of the hypothesis that the PAS domain and/or *N–*terminal α–helix bind to the cyclic–nucleotide binding domain will require expression and purification of the cyclic–nucleotide binding domain.

## Supporting Information

Figure S1 **^15^N-HSQC spectrum of hERG** ***N*** **–terminal domain (M1-K135).** The spectrum was collected at 25°C on a Bruker 900 MHz Avance II spectrometer fitted with a cryoprobe. Backbone amide proton resonances are identified by their residue numbers. Sidechain N-H resonances are indicated by an sc suffix.
(EPS)

Figure S2 **Amino acid sequence of hERG PAS domain and overview of NMR data.** The secondary structure elements are labelled according to the hERG PAS domain PDB structure (2L0W). Hydrogen bonds constraints used in the structure calculation are indicated as black circles. Chemical shift index (CSI) prediction of the secondary structure is shown immediately above the amino acid sequence. Thick and thin bars indicate strong and weak NOE cross-peaks intensities for the sequential proton–proton NOE connectivities (*d*NN, *d*αN and *d*βN). The observed medium-range NOEs *d*NN(*i*, *i*+2), *d*αN(*i*, *i*+2), *d*αN(*i*, *i*+3), *d*αβ(*i*, *i*+3) and *d*αN(*i*, *i*+4) and are indicated by lines connecting the two residues that are related by the NOE.
(EPS)

Figure S3 **Relative amplitudes of τfast and τslow that comprise deactivation rates.** Tail currents recorded over a range of potentials (Vm) following a test pulse to +40 mV are fit with a double exponential function (see methods). Relative amounts of τfast and τslow components are then plotted against voltage. At negative potentials, where τfast dominates, there is little difference in relative amplitudes between WT (black) and mutant channels (Δ2-9: blue; Δ2-25: green; GGSmut: red). However, at less negative potentials (>−90 mV) there is a significant increase in the relative amounts of τfast in mutant channels (Δ2-9, Δ2-25, GGSmut) compared to WT hERG.
(EPS)

Table S1 **Time constants for fast component of deactivation, at −30 kJ mol^−1^, for WT and all mutants investigated in this study.**
(DOC)

Table S2 **Time constants for fast and slow components of deactivation and ratio of fast and slow components of deactivation, at -120 mV, for WT and all mutants investigated in this study.**
(DOC)
