# Supplementary material for: The N–Terminal Tail of hERG Contains an Amphipathic α–Helix That Regulates Channel Deactivation
Source: PLoS One. 2011 Jan 13;6(1):e16191. doi: 10.1371/journal.pone.0016191 (PMC3020963; doi:10.1371/journal.pone.0016191)
Supplement: Table S1 — Time constants for fast component of deactivation, at −30 kJ mol−1, for WT and all mutants investigated in this study. (DOC) [file pone.0016191.s004.doc]

**Table S1:** Time constants for fast component of deactivation, at -30 kJ mol^-1^, for WT and all mutants investigated in this study.

|  | n | V_0.5_  (mV) | Slope  (mV) | z_g_ (e-) | ΔG_0_  (kJmol^-1^) | τ_fast_ at -30 kJmol^-1^  (ms) |
| --- | --- | --- | --- | --- | --- | --- |
| WT | 11 | -23.2 ± 0.8 | 8.4 ± 0.3 | 3.0 ± 0.1 | -6.8 ± 0.2 | 28.5 ± 2.8 |
| Δ2-9 | 14 | -5.6 ± 0.9 | 8.3 ± 0.4 | 2.9 ± 0. | -1.9 ± 0.3 | 13.3 ± 1.0 |
| Δ2-25 | 5 | -22.7 ± 0.7 | 8.4 ± 0.6 | 3.0 ± 0.1 | -6.6 ± 0.5 | 7.5 ± 0.8 |
| GGS | 10 | -18.5 ± 0.6 | 6.8 ± 0.5 | 3.7 ± 0.2 | -6.8 ± 0.6 | 11.0 ± 1.1 |
| P2A | 7 | -22.3 ± 1.0 | 6.3 ± 0.1 | 3.8 ± 0.1 | -8.3 ± 0.4 | 20.7 ± 1.1 |
| V3A | 8 | -19.7 ± 0.6 | 6.4 ± 0.2 | 3.8 ± 0.1 | -7.3 ± 0.3 | 24.7 ± 0.8 |
| R4A | 6 | -19.3 ± 0.3 | 7.5 ± 0.2 | 3.3 ± 0.1 | -6.2 ± 0.2 | 22.9 ± 1.0 |
| R5A | 8 | -16.4 ± 0.5 | 7.1 ± 0.4 | 3.5 ± 0.2 | -5.6 ± 0.3 | 16.5 ± 1.0 |
| G6A | 7 | -17.3 ± 0.6 | 7.6 ± 0.6 | 3.3 ± 0.3 | -5.5 ± 0.8 | 12.7 ± 1.3 |
| H7A | 8 | -18.5 ± 0.7 | 7.7 ± 0.4 | 3.3 ± 0.1 | -5.9 ± 0.4 | 21.2 ± 1.9 |
| V8A | 8 | -20.9 ± 0.6 | 7.7 ± 0.3 | 3.3 ± 0.1 | -6.6 ± 0.4 | 24.7 ± 1.6 |
| A9V | 6 | -18.9 ± 0.5 | 7.4 ± 0.3 | 3.3 ± 0.1 | -6.1 ± 0.3 | 24.9 ± 0.7 |
| P10A | 14 | -17.5 ± 0.6 | 7.6 ± 0.3 | 3.3 ± 0.1 | -5.6 ± 0.3 | 44.4 ± 2.4 |
| Q11A | 11 | -22.8 ± 0.8 | 7.5 ± 0.2 | 3.3 ± 0.1 | -7.3 ± 0.3 | 33.5 ± 1.5 |
| N12A | 8 | -18.6 ± 0.6 | 7.8 ± 0.7 | 3.4 ± 0.1 | -6.3 ± 0.3 | 19.9 ± 1.3 |
| T13A | 15 | -27.6 ± 0.7 | 7.9 ± 0.2 | 3.0 ± 0.1 | -8.2 ± 0.3 | 38.2 ± 2.2 |
| F14A | 8 | -27.2 ± 0.9 | 8.3 ± 0.5 | 3.0 ± 0.1 | -8.1 ± 0.6 | 24.0 ± 2.1 |
| L15A | 7 | -22.7 ± 0.6 | 7.9 ± 0.3 | 3.2 ± 0.1 | -7.0 ± 0.3 | 30.6 ± 0.9 |
| D16A | 10 | -21.2 ± 0.7 | 7.4 ± 0.1 | 3.2 ± 0.1 | -6.7 ± 0.3 | 54.2 ± 4.7 |
| T17A | 14 | -22.1 ± 0.7 | 6.8 ± 0.1 | 3.4 ± 0.1 | -7.6 ± 0.3 | 35.3 ± 2.8 |
| I18A | 12 | -24.3 ± 0.6 | 6.2 ± 0.1 | 3.9 ± 0.1 | -9.3 ± 0.3 | 33.0 ± 1.7 |
| I19A | 13 | -25.1 ± 0.6 | 7.8 ± 0.2 | 3.2 ± 0.1 | -7.7 ± 0.5 | 19.2 ± 1.3 |
| R20A | 20 | -27.0 ± 0.6 | 7.8 ± 0.2 | 3.2 ± 0.1 | -8.4 ± 0.3 | 20.4 ± 1.0 |
| K21A | 11 | -26.1 ± 0.7 | 8.3 ± 0.4 | 3.1 ± 0.1 | -7.9 ± 0.3 | 22.6 ± 1.3 |
| F22A | 11 | -25.3 ± 1.0 | 8.4 ± 0.4 | 3.0 ± 0.1 | -7.5 ± 0.5 | 26.5 ± 2.1 |
| E23A | 7 | -27.6 ± 0.6 | 7.8 ± 0.3 | 3.3 ± 0.1 | -8.7 ± 0.5 | 28.6 ± 3.4 |
