# Supplementary material for: The N–Terminal Tail of hERG Contains an Amphipathic α–Helix That Regulates Channel Deactivation
Source: PLoS One. 2011 Jan 13;6(1):e16191. doi: 10.1371/journal.pone.0016191 (PMC3020963; doi:10.1371/journal.pone.0016191)
Supplement: Table S2 — Time constants for fast and slow components of deactivation and ratio of fast and slow components of deactivation, at -120 mV, for WT and all mutants investigated in this study. (DOC) [file pone.0016191.s005.doc]

**Table S2:** Time constants for fast and slow components of deactivation and ratio of fast and slow components of deactivation, at ‑120 mV, for WT and all mutants investigated in this study.

|  | n | τ_fast-120_  (ms) | τ_slow-120_  (ms) | Ratio  (A_fast_ / A_fast_+A_slow_) |
| --- | --- | --- | --- | --- |
| WT | 11 | 33.7 ± 1.0 | 185.6 ± 7.2 | 0.83 ± 0.004 |
| Δ2-9 | 14 | 11.4 ± 0.3 | 92.3 ± 7.1 | 0.83 ± 0.005 |
| Δ2-25 | 5 | 8.7 ± 0.3 | 65.0 ± 3.4 | 0.84 ± 0.01 |
| GGS | 10 | 7.5 ± 0.2 | 79.3 ± 5.1 | 0.90 ± 0.01 |
| P2A | 7 | 14.9 ± 0.5 | 156.4 ± 7.6 | 0.81 ± 0.01 |
| V3A | 8 | 17.6 ± 0.4 | 166.7 ± 4.8 | 0.83 ± 0.004 |
| R4A | 6 | 19.6 ± 0.5 | 163.5 ± 8.6 | 0.84 ± 0.01 |
| R5A | 8 | 12.9 ± 0.3 | 134.6 ± 3.6 | 0.86 ± 0.01 |
| G6A | 7 | 11.9 ± 0.7 | 141.1 ± 11.8 | 0.85 ± 0.01 |
| H7A | 8 | 17.7 ± 0.7 | 174.8 ± 9.0 | 0.85 ± 0.004 |
| V8A | 8 | 22.4 ± 0.8 | 164.7 ± 7.5 | 0.86 ± 0.004 |
| A9V | 6 | 20.8 ± 0.7 | 176.3 ± 9.9 | 0.85 ± 0.01 |
| P10A | 14 | 36.9 ± 1.0 | 255.3 ± 8.4 | 0.85 ± 0.01 |
| Q11A | 11 | 31.1 ± 1.1 | 198.9 ± 5.6 | 0.81 ± 0.002 |
| N12A | 8 | 15.5 ± 0.4 | 158.6 ± 8.0 | 0.86 ± 0.003 |
| T13A | 15 | 52.3 ± 2.1 | 304.5 ± 8.9 | 0.79 ± 0.01 |
| F14A | 8 | 30.6 ± 1.3 | 194.3 ± 4.3 | 0.87 ± 0.01 |
| L15A | 7 | 31.0 ± 1.6 | 203.7 ± 8.6 | 0.84 ± 0.01 |
| D16A | 10 | 52.6 ± 2.7 | 277.0 ± 10.9 | 0.73 ± 0.01 |
| T17A | 14 | 27.3 ± 1.0 | 240.8 ± 16.5 | 0.86 ± 0.01 |
| I18A | 12 | 19.9 ± 0.5 | 206.6 ± 15.9 | 0.89 ± 0.01 |
| I19A | 13 | 19.9 ± 0.9 | 153.6 ± 12.3 | 0.92 ± 0.01 |
| R20A | 20 | 22.0 ± 0.7 | 182.9 ± 10.9 | 0.9 ± 0.01 |
| K21A | 11 | 26.5 ± 0.6 | 182.9 ± 10.9 | 0.86 ± 0.01 |
| F22A | 11 | 31.9 ± 1.5 | 230.9 ± 11.2 | 0.89 ± 0.01 |
| E23A | 7 | 30.0 ± 1.4 | 239.6 ± 9.5 | 0.85 ± 0.01 |
